# Testing a dominance-region hypothesis for interaural time discrimination using off-frequency maskers

**DOI:** 10.1121/10.0042819

**Published:** 2026-03-13

**Authors:** Richard L. Freyman, Benjamin H. Zobel, Patrick M. Zurek

**Affiliations:** 1 Department of Speech, Language, and Hearing Sciences, University of Massachusetts Amherst, Amherst, Massachusetts 01003 USA; 2 Sensimetrics Corporation, Gloucester, Massachusetts 01930 USA

## Abstract

This study evaluated a recent proposal that the steep roll-off in fine structure–based sensitivity to interaural time delay (ITD-FS) between 1.2 and 1.5 kHz could be because of diminishing spread of excitation to a dominance region around 700 Hz. Experiment 1 measured the effects of interaurally uncorrelated masking noise lowpass filtered at 800 Hz on ITD-FS sensitivity for higher-frequency tones. Experiment 2 investigated the complementary configuration, with tones lower in frequency than the masking noise. In neither experiment was it found that masking the region around 700 Hz was especially effective in reducing ITD-FS discriminability at other frequencies.

## 1. Introduction

Arrival time differences between the ears create two classes of cues for sound localization and in-head lateralization that dominate in two roughly non-overlapping frequency regions. Sensitivity to the interaural time delay in waveform fine structure (ITD-FS) is restricted to below about 1500 Hz (Klumpp and Eady, 1956). Sensitivity to interaural delay in the stimulus envelope, because of onset delays or delays in envelope modulation, exists across the entire frequency range of hearing but is more important in the higher-frequency region. The current study concerns a curious feature of fine structure interaural delay sensitivity, which is that although it is acute up to about 1200 Hz, it all but disappears by 1500 Hz. This rapid decline and loss of sensitivity to ITD-FS in a span of only 200–300 Hz has been a long-standing puzzle in auditory research (Brughera *et al.*, 2013; Klug and Dietz, 2022).

Recently, Goupell *et al.* (2024) proposed a novel explanation for the sharp decline in ITD-FS sensitivity. The explanation is based in part on the well-established dominance region peaking at around 700 Hz for the processing of interaural time differences and localization in general (e.g., Bilsen and Raatgever, 1973; Wightman and Kistler, 1992; Tollin and Henning, 1999; Macpherson and Middlebrooks, 2002; Folkerts and Stecker, 2022). Goupell *et al.* (2024) suggested that discrimination performance at frequencies above 700 Hz could be mostly or entirely the result of cochlear-based downward spread of excitation to neurons tuned to around 700 Hz where interaural delay sensitivity is greatest. The authors posit that the reason that ITD-FS performance falls off so sharply above 1200 Hz is not because neurons tuned to these frequencies become dramatically less sensitive to interaural delay. Instead, it is that downward spread of cochlear excitation rolls off exceptionally steeply, as has been well known for many decades (see Whiteford *et al.*, 2020, for a recent psychoacoustical example). Tones of 1500 Hz and above would be expected to produce little if any excitation of neurons tuned to 700 Hz, which, according to this explanation, is the reason for the ITD-FS insensitivity at those higher frequencies. Goupell *et al.* (2024) measured ITD-FS discrimination performance as a function of frequency and level and obtained findings that were consistent with this peripheral filtering explanation. Specifically, the decrease in discrimination performance as a function of level at those higher frequencies was in line with predicted decreases in downward spread of excitation as level was reduced.

This explanation makes testable predictions of the effects of introducing masking noise to render ITD-sensitive neurons unavailable. If, according to one version of the theory, the regions at and below 700 Hz are collectively responsible for all ITD-FS discrimination, then masking those sensitive regions with lowpass-filtered noise should render ITD-FS discrimination at higher frequencies all but impossible. This was tested in experiment 1 of the current study. If, according to a stronger version of the theory, it is the case that *only* the 700 Hz region is responsible for ITD-FS discrimination (as opposed to 700 Hz and below), then masking the region around 700 Hz should also strongly affect discrimination at frequencies below 700 Hz. This was explored in experiment 2.

## 2. Experiment 1. ITD-FS discrimination from 1.0 to 1.5 kHz in quiet and in noise

This experiment investigated fine structure ITD discrimination at 1.0–1.5 kHz both in quiet and in the presence of lowpass-filtered noise intended to mask downward spread of excitation to regions ≤800 Hz. If these regions were entirely responsible for ITD processing, then the presence of masking noise would be expected to severely impair discrimination.

### 2.1 Method

ITD-FS discrimination was tested with a two-interval forced choice (2IFC) task. A single tone was presented in each interval at the same frequency, and participants responded on a touch pad to indicate whether the tone in the second interval was presented to the left or right of the tone in the first interval. Tones were sinusoids ranging from 1.0 to 1.5 kHz in 100 Hz steps and were 500 ms in duration, including 100 ms raised cosine onset and offset ramps. Fine structure ITDs of 80 *μ*s favoring the left or right ears were imposed on the tones by computing a two-channel sine wave with the required phase delay to one channel in one interval and the same phase delay to the opposite channel in the other interval, with the interval order determined at random on each trial. Thus, across the two intervals of a trial, there was a 160-*μ*s ITD in the fine structure. There was no onset delay; the left and right channels were ramped on and off synchronously.

Noises were created by convolving a Gaussian noise with a windowed-sinc filter (Hanning window duration = 20 ms, high-side slope > 150 dB/octave). In experiment 1, the noise was lowpass filtered at 800 Hz. Each interval of a two-interval trial that included noise consisted of 900 ms of noise, with the tone beginning 200 ms after the noise onset and ending 200 ms before the noise offset. There were 500 ms in between tone presentations across the two intervals, and therefore 100 ms of silence between the noise in intervals 1 and 2 when it was present. When noise was presented, the signal-to-noise spectrum level ratio (S/No) was 23 dB. The justification for this choice of S/No will be discussed later in this paper.

**Fig. 1.**
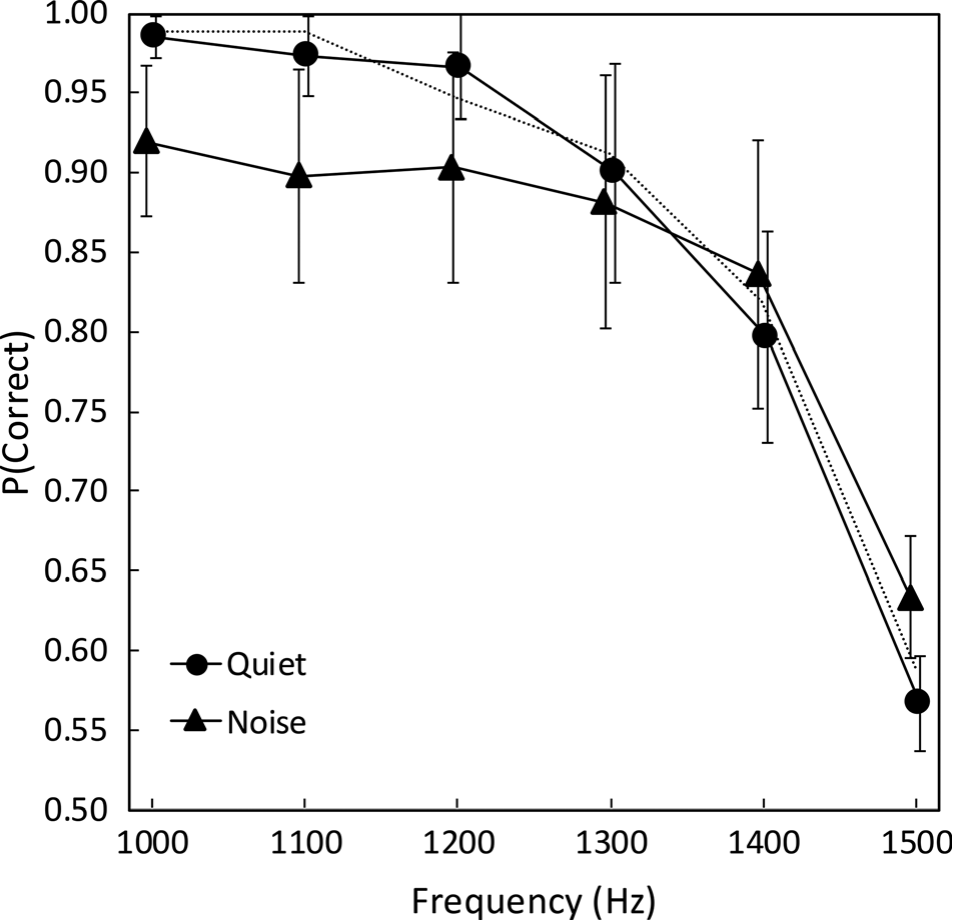
Average ITD-FS discrimination performance for six subjects for pure tones of 1.0–1.5 kHz with ITD-FS = ±80 *μ*s. Filled circles show the data in quiet, and triangles show the data in noise, with slight horizontal staggering to improve visualization. The vertical lines show ±1 standard error of the mean. The light dashed line shows a repetition of the quiet condition with the tone level lowered by 7 dB to equate the signal power in the quiet and noise conditions. P = proportion.

The stimuli were generated in matlab software and delivered via a Behringer U-Phoria UMC202HD audio interface (Dubai, United Arab Emirates) at a 192 kHz sampling rate to Sennheiser HD 280 Pro headphones (Wedemark, Germany). When presented in quiet, the level of the tone was 69 dB sound pressure level (SPL) at each ear. When presented in noise, the tone level was 7 dB lower, helping to preserve a similar overall sound level of tone-alone and tone-plus-noise trials. To ensure that this decrease in tone level did not affect ITD performance, the experiment was repeated with the tones in quiet at 62 dB SPL, with no meaningful difference in results (see Fig. 1). It is noted that level effects reported by Goupell *et al.* (2024) formed a substantial portion of the evidence supporting their hypotheses; but here, the levels of 69 and 62 SPL were clearly too similar or too high to observe any effect of the 7 dB difference.

Stimuli were presented in blocks of 20 trials with a fixed frequency and fixed noise condition. Blocks for all six frequencies were completed in random order, with this procedure repeated after all conditions had been visited once. For each frequency, blocks with three different noise conditions—tone in quiet, tone in interaurally uncorrelated noise, and tone in diotic noise—were completed in random order before moving to the next frequency. Five blocks per frequency/noise condition were completed for a total of 100 trials per data point for each of 6 young, experienced listeners (4 female, 20–22 yrs) with normal hearing (thresholds ≤20 dB HL from 250 through 8000 Hz). The diotic-noise data are not presented in this paper as it quickly became clear that performance was unexpectedly much better in the noise than in quiet (e.g., 75% correct at 1500 Hz in the diotic noise). Follow-up work with lowpass diotic noise just below the signal frequency produced an even stronger enhancement effect. The effect was traced to interactions between tone and asymmetrically placed noise that created large envelope delays. A full exploration of these interactions is beyond the scope of this paper.

### 2.2 Results and discussion

Figure 1 (circles) shows average ITD-FS discrimination performance (proportion correct) as a function of frequency in the quiet condition. Performance stayed above 90% correct through 1300 Hz and declined to below 60% by 1500 Hz. This drop is consistent with the steepness of the roll-off in Goupell *et al.* (2024) of between 30 and 40 percentage points in the span of only 200 Hz, although the steep portion of the average function in that study was between 1200 and 1400 Hz, which is a lower range than ours by about 100 Hz.

The triangles in Fig. 1 show performance in the presence of uncorrelated noise. Through 1200 Hz, the reduction in performance in the noise condition was fairly consistent but modest, ranging from 6 to 8 percentage points. At 1300 Hz and above, the difference was much smaller or even slightly in the opposite direction. It is clear that the steeply declining performance in the high frequencies was not accelerated by the presence of the 800 Hz lowpass noise.

It is important to assess whether the noise employed in this study was sufficient to obscure any contribution from the 700 Hz region via downward spread of excitation from the tested frequencies. To answer this question, we considered the detectability of a 700 Hz tone in the noise used here. The critical S/No ratio for detecting a monaural 700 Hz tone in monaural noise is approximately 16–17 dB (Hawkins and Stevens, 1950), so we would expect a diotic 700 Hz tone, presented at 23 dB S/No in diotic noise, to be only 6–7 dB above threshold in the noise. The stimuli in experiment 1 were presented with an ITD-FS of 80 *μ*s in an interaurally uncorrelated noise, a condition similar to that which produced a masking release of about 4 dB (Rabiner *et al.*, 1966). Starting with 6–7 dB sensation level (SL) from the critical ratio estimate and adding ∼4 dB more for potential binaural masking release, it was estimated that a 700 Hz tone with an 80 *μ*s interaural delay would be ∼10 dB SL at the 23 dB S/No noise employed and therefore should have been effectively inaudible (with indiscriminable ITDs) at an S/No much below 13 dB.

To confirm this prediction, we tested ITD-FS performance at 700 Hz for ITD-FS = ±80 *μ*s using the same subjects, stimulus levels, and procedures, but with S/No varying in 5 dB steps. Mean performance was 87, 63, 56, 53, and 51% correct at 23, 18, 13, 8, and 3 dB S/No respectively. The calculation of 13 dB S/No as the approximate limit for ITD-FS discrimination was found to be a reasonable estimate (56% correct), although even 18 dB produced only 63% correct performance. The 1.0–1.5 kHz tones from experiment 1 were presented at S/No = 23 dB, only 10 dB higher than this 13 dB value. It follows that those tones would be able to contribute to ITD-FS discrimination through spread of activity to the 700 Hz region only if it was effectively attenuated by no more than 5–10 dB through downward spread of excitation. The literature suggests downward excitation pattern slopes on the order of 100 dB/octave (Whiteford *et al.*, 2020). This steepness of attenuation in combination with the low SL of even a 700 Hz tone in the noise, leads to confidence that the masking noise made regions ≤700 Hz unavailable to excitation from tones in the 1.0–1.5 kHz region.

## 3. Experiment 2. Effects of higher-frequency masking on lower-frequency ITD-FS discrimination

Having found little evidence that downward spread to 700 Hz and below supports ITD-FS at higher frequencies, we next examined a prediction of the more extreme version of the dominance region theory proposed by Goupell *et al.* (2024). If there is only a single dominant channel at 700 Hz, then masking that region should also degrade ITD-FS discrimination for lower frequencies. Experiment 2 tested this prediction. Procedures were the same as in experiment 1 with one notable difference: Instead of fixing the fine structure ITD at 80 *μ*s, six different delays (determined by pilot testing) were tested in each condition to obtain psychometric functions. Each ITD-FS was visited once before repeating to complete five series of 20-trial blocks, for a total of 100 trials per data point per subject. When noise was present, it was always interaurally uncorrelated and presented at 23 dB S/No. The same six subjects from experiment 1 contributed the data for experiment 2.

### 3.1 Results and discussion

**Fig. 2.**
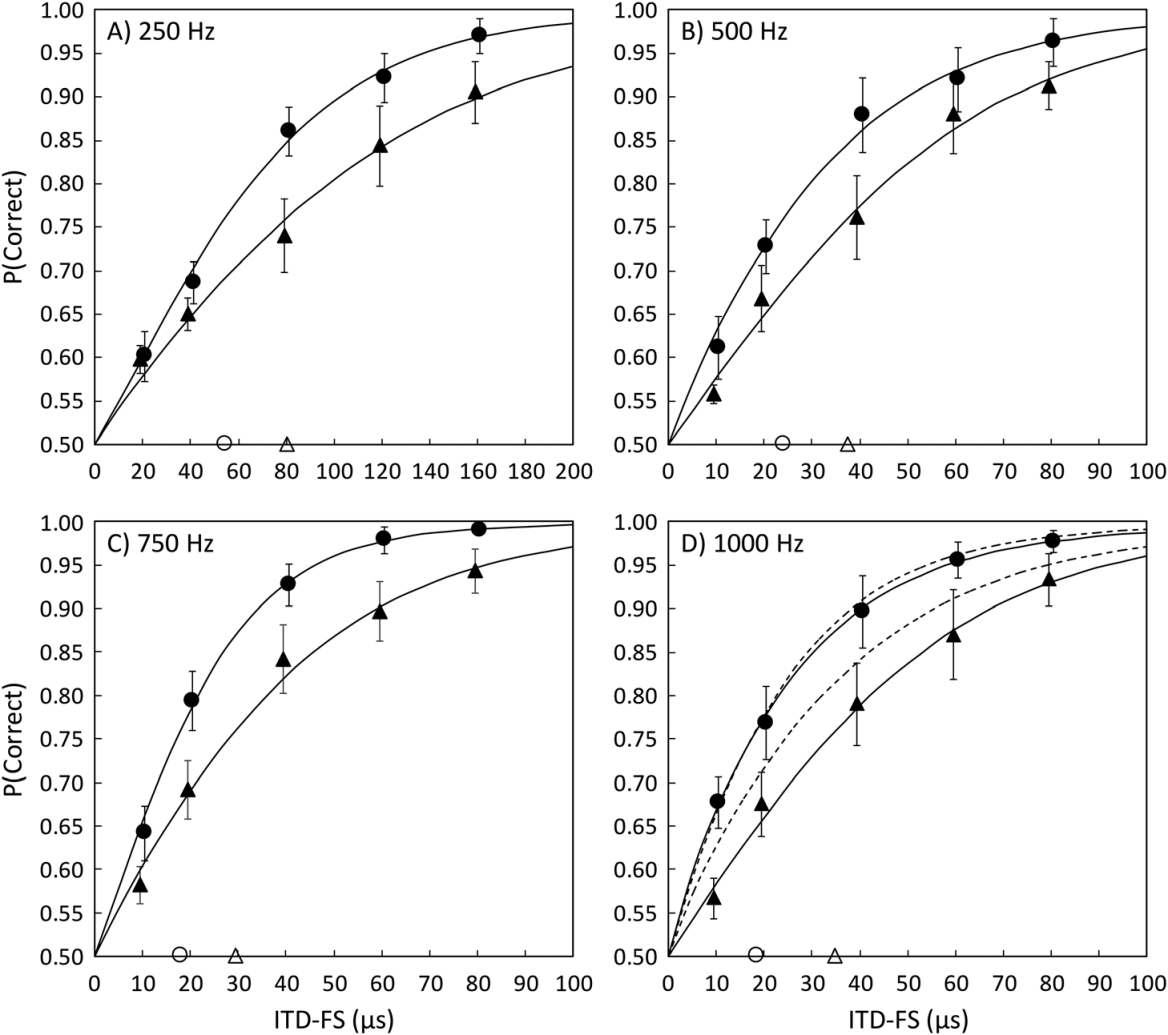
Pooled psychometric functions across six subjects showing performance in quiet (filled circles) and in the presence of uncorrelated noise (filled triangles). Thresholds for a d′ of 1.0 are shown with the open circles and triangles on the abscissa. Vertical lines depict ±1 standard error of the mean, with a slight horizontal staggering to enhance visualization. The details of the noise conditions as well as the numerical thresholds are given in Table 1. (A) Note that the abscissa has a different scale from the other three panels. In (D) the 1000 Hz ERB condition (see text) in quiet and noise is displayed by the light dashed lines. P = proportion.

Pooled psychometric functions from the six subjects are shown in Fig. 2(A) for a 250 Hz tone in quiet and with the noise passband from 600 to 1400 Hz. The quiet condition is shown with the filled circles and the noise condition with the filled triangles. The thresholds determined from the fitted functions (corresponding to d′ = 1.0) are indicated by the open circle (quiet) and triangle (noise) on the abscissa. The effect of noise is easily visible, although sensitivity is clearly not eliminated by noise. Thresholds for d′ = 1 were 54.3 *μ*s in quiet and 80.2 *μ*s in noise, a relative change (noise threshold/quiet threshold) of 1.48 (see Table 1, top row).

**Table 1.** Experiment 2 results.

Tone frequency (Hz)	Noise passband (Hz)	ITD-FS threshold in quiet (*μ*s)	ITD-FS threshold in noise (*μ*s)	Relative effect noise/quiet
250	600–1400	54.3	80.2	1.48
500	850–1650	24.0	37.4	1.56
750	1100–1900	17.9	29.4	1.64
1000	1350–2150	18.4	34.8	1.89
1000 ERB	1900–2700	18.3	25.8	1.41

The same detectability analysis conducted for experiment 1 was done to test the masking adequacy of the noise. That analysis suggests that anything more than 5–10 dB of attenuation from 250 Hz in the upward spread of excitation would render the 700 Hz region unavailable. Although the slopes of the high-frequency spread of excitation are shallower than the low-frequency slopes, they are far greater than 5–10 dB in a span of almost 1.5 octaves from 250 to 700 Hz (e.g., Whiteford *et al.*, 2020). The results shown in Fig. 2(A) indicate that masking the 700 Hz region had some effect, but it was not of sufficient magnitude to indicate that ITD-FS discrimination at 250 Hz depended *entirely* on upward spread of excitation to the 700 Hz region.

Additional conditions were run to explore the relative importance of masking the 700 Hz region compared to other regions. In these conditions both the tone and the masking noise band were raised in three 250 Hz increments (see first four rows of Table 1). The purpose was to keep the frequency relationship of the tone to the masker passband the same but change the region being masked from that which included the posited region of dominance (e.g., 600–1400 Hz) to a region entirely beyond (1350–2150 Hz). The results with these conditions, shown in Fig. 2, exhibit trends like those seen with the 250 Hz tone. There appears to be a generalized effect of introducing noise, not one that depends on the masking of an exceptionally sensitive region. In fact, as the masking noise passband moved above the dominance region, the relative change in threshold actually increased gradually from 1.48 to 1.89 times the threshold in quiet.

Regarding that trend, one issue to consider is that as the frequency was raised across these conditions, the proximity of the noise to the tone was kept the same in Hz, but a spacing based on log frequency or auditory filter width may be more appropriate. Therefore, as shown in the bottom row of Table 1, an additional condition was run to equate proximity in equivalent rectangular bandwidth (ERB) units (Glasberg and Moore, 1990). Subjects discriminated fine structure ITDs at 1000 Hz while the passband of the masker was positioned with its lower edge 5.1 ERB units away (1900 Hz)—the same ERB distance as 600 Hz is to 250 Hz (Glasberg and Moore, 1990). The noise bandwidth remained the same at 800 Hz. The quiet condition was repeated and interleaved with the noise during data collection as it was with the other conditions. The quiet (upper) and noise (lower) fitted functions are shown by the light dashed lines in Fig. 2(D). The data from the repeated quiet condition closely matched the initial 1000 Hz quiet data, demonstrating the stability of those data in this group of listeners. The presence of the 1900–2700 Hz noise was about half as effective in reducing performance as was the 1350–2150 Hz noise. As shown in Table 1, threshold was increased by a factor of 1.41 in this new noise condition, similar to the factor of 1.48 observed for 250 Hz with the same ERB spacing of the noise.

## 4. Discussion

The modest increase in the ITD-FS threshold that we observed from the introduction of noise in these two experiments is not unexpected. For example, there is an extensive literature showing that an uninformative interferer, often diotic, at 500 Hz, can degrade interaural delay discrimination performance for a signal at 4 kHz (e.g., McFadden and Pasanen, 1976; Heller and Richards, 2010). In addition to the larger frequency separation in those studies, they also usually present target and interferer gated on together. Bernstein and Trahiotis (1995), however, still measured interference effects when the interference was continuous and interaurally uncorrelated. The results of these types of studies have been interpreted as reflecting a generalized effect resulting from a degree of obligatory integration in the central auditory system, as opposed to monaural interactions (e.g., Bernstein and Trahiotis, 1995).

Other studies have focused on conditions where the noise covered frequency regions closer to the signal frequency, with the express purpose of masking “off-frequency listening” (e.g., Dreyer and Oxenham, 2008; Bernstein and Trahiotis, 2008; and the current study). In these cases it is suggested that noise masks the spread of excitation to peripheral neurons, which when unmasked add informative input to the binaural system. Goupell *et al.* (2024) considered the possibility that ITD discrimination performance between 1.0 and 1.5 kHz might be entirely dependent on off-frequency listening and specifically that performance fades abruptly over that range because downward spread of excitation to the dominant region around 700 Hz fades abruptly.

Results from detection studies, where broadband noise covers the signal frequency in the 1.0–1.5 kHz region (e.g., Hirsh and Burgeat, 1958), likewise suggest the necessity of on-frequency processing. Listening through an off-frequency channel would provide a much lower signal-to-noise ratio than that available at the signal frequency itself. In addition to these psychoacoustic data, there is ample physiological evidence that ITD sensitivity is available through a broad frequency range and is not confined to a narrow channel (Joris and Yin, 2007).

Although we cannot be sure of the origin of the interference effects observed in our study, it is interesting to note that in our pooled data we observed at least some interference (of more than a few percentage points) in virtually all the conditions tested except for those in experiment 1 with tones from 1.3 to 1.5 kHz and a noise lowpass filtered at 800 Hz. This could be an indication that when the noise did produce a modest effect (e.g., from 1.0 to 1.2 kHz) it was either because of the upward spread of excitation of the noise into the filters tuned to the signal frequency or because the noise masked off-frequency listening. Considering the latter possibility, the absence of an effect from 1.3 to 1.5 kHz may have been because steep peripheral filtering prevented any contribution from units tuned to the region of the noise passband, whether the noise was present or not. Under that interpretation, we would agree with Goupell *et al.* (2024) that the frequencies from 1.3 to 1.5 kHz become too distant for the spread of excitation to reach the 700 Hz region, given the steep slope of the downward spread of excitation. The main difference is that our data do not suggest that the 700 Hz region plays a uniquely important or dominant role.

A final aspect of this study worth repeating is the fact that we exclusively used uncorrelated noise to mask off-frequency listening. As mentioned in Sec. 2.1, we discovered that diotic noise asymmetrically placed relative to the signal frequency produced enhancement effects in the conditions of experiment 1, which we suspect were because of signal-masker interactions creating large interaural envelope disparities. We cannot be entirely certain that uncorrelated noise did not produce some smaller enhancement, as hinted at by the results at 1400 and 1500 Hz in Fig. 1, where mean performance was slightly better in the noise condition compared to the quiet condition. We do not know the origin of these smaller effects, if they exist, and are not concerned that they would affect any of the conclusions of this study.

## 5. Summary and conclusions

The frequency region near 700 Hz has been shown to be most sensitive for ITD-FS discrimination and dominant for sound localization in general. The current study tested the notion, proposed by Goupell *et al.* (2024), that ITD-FS discrimination at other frequencies relies on spread of excitation to the 700 Hz region. If so, it could help explain the sudden loss of sensitivity to ITD-FS as frequency increases from 1200 to 1500 Hz, because downward spread of excitation measured psychoacoustically and physiologically in the auditory periphery is also exceptionally steep (see Goupell *et al.*, 2024, for details of this explanation). In experiment 1 we introduced interaurally uncorrelated masking noise sufficient to mask the spread of excitation to 700 Hz. However, this caused no worsening of ITD-FS discrimination at frequencies along the steep edge of decline in sensitivity in the current study (1300–1500 Hz). This suggests that the steep decline in sensitivity across these higher frequencies is likely to be based on mechanisms other than diminishing downward spread of excitation to the 700 Hz region.

In a parallel study, experiment 2 assessed the extent to which ITD-FS sensitivity at low frequencies was assisted by upward spread of excitation to units tuned to 700 Hz. Introducing masking noise that included the 700 Hz region decreased sensitivity for a 250 Hz tone by a modest amount. However, repeating the experiment while shifting the tone and masking noise upward in frequency produced similar proportional increases in threshold and did not indicate the 700 Hz region to be unusually effective relative to other regions. Together, the results of the two experiments appear to be inconsistent with a hypothesis that ITD-FS sensitivity across frequencies is mediated primarily by spread of excitation to the 700 Hz region.

## Data Availability

The data collected in the reported studies are available from the corresponding author upon request.
